# Brain Metastasis From Prostate Adenocarcinoma: Case Report and Review of Literature

**DOI:** 10.4021/wjon442w

**Published:** 2012-04-23

**Authors:** Graziano Taddei, Sara Marzi, Gino Coletti, Danilo De Paulis, Alessandro Ricci, Renato J. Galzio

**Affiliations:** a University of L’Aquila, Italy; b Division of Neurosurgery, S. Salvatore Hospital, L’Aquila, Italy; c Division of Pathology, S. Salvatore Hospital, L’Aquila, Italy; d Department of Health Sciences, University of L’Aquila, Italy

**Keywords:** Prostate adenocarcinoma, Brain metastasis, Small cells prostate carcinoma

## Abstract

It is rare for prostate carcinoma to metastasize to the central nervous system. It often represents a terminal event with death in one year frequently due to the advanced systemic disease. Starting by a case report, we also reviewed the relevant literature to focus on this uncommon entity from epidemiology to clinical manifestation and therapeutic strategies. In this article, a case of multiple brain prostate metastasis is reported and a review of relevant literature is also discussed. Treatments available for intracranial metastasis include neurosurgery, external beam radiation and hormonal manipulation. Surgery associated with whole brain radiotherapy seems to be effective in the control of brain lesions both relieving neurological symptoms and prolonging survival, even if prognosis remains dismal. From this case, we concluded that brain metastasis from prostate carcinoma is a rare, terminal event with death in one year frequently due to the advanced systemic disease. A better understanding of the biology of prostate carcinoma will help clarify the basis for its metastasis to the brain.

## Introduction

Brain metastasis occurs in approximately 25% of patients with malignant disease, and, conversely, 50% of neoplasms in the brain are metastatic [1]. The most common sources of metastasis to the brain are carcinoma of the lung, breast, kidney, and melanoma [2, 3]. It is rare for prostate carcinoma to metastasize to the central nervous system (CNS) [1]. The frequency of brain metastasis reported in autopsy series is very low in patients with a pre-mortem diagnosis of prostate carcinoma [1], and antemortem diagnosis of intracranial metastasis has been achieved in only 0.1% of cases [4]. Starting by a case report, a review of relevant literature is discussed to better understand this uncommon entity.

## Case Report

**Figure 1:**
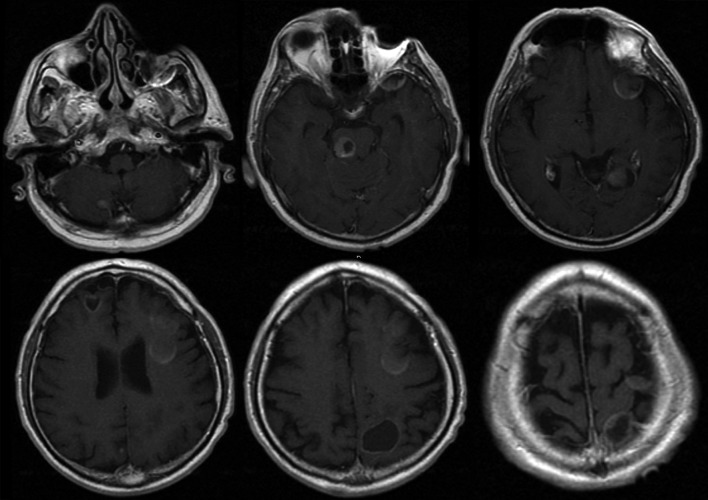
Brain MR with gadolinium showing the six distinct intraparenchymal lesions.

**Figure 2:**
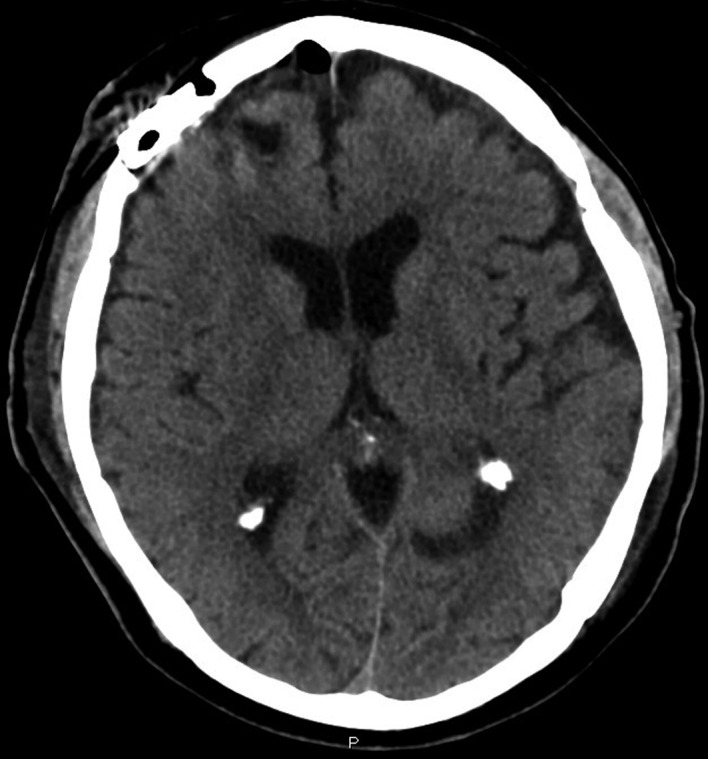
Post-operative brain TC.

**Figure 3:**
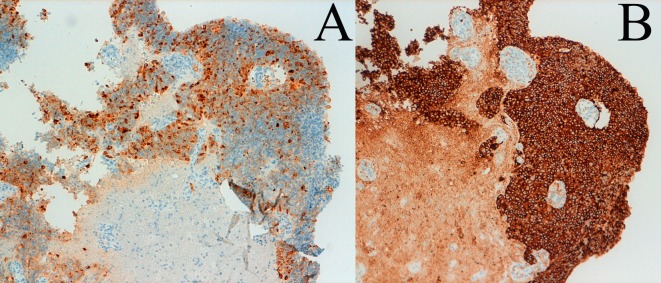
Histologic patterns: immunohistochemical stain for PSA (A) and PSMA (B).

A man, 62 years old, was admitted at our Institute on October 2010 with a recent history of left limbs hypoesthesia and gait instability. Moreover, at the neurological examination a complete cerebellar syndrome associated to hypogeusia and weakness of left arm were found. A brain Magnetic Resonance (MR) with gadolinium showed six distinct cerebral cystic lesions (Fig. 1). A total-body Computed Tomography (CT) was performed with the evidence of multiple right lung nodules, a cystic right renal lesion and irregular prostate margins. The patient did not refer urinary impairment and the serum PSA value was 38.12 ng/dl. Multiple prostate biopsies were obtained and the histology showed adenocarcinoma and a positive immunohistochemical stain for PSA. The right frontal lesion was removed to achieve histologic characterization (Fig. 2). It was demonstrated to be a metastatic prostatic adenocarcinoma with a diffuse immunohistochemical stain for PSA and PSMA (Fig. 3). The patient underwent palliative radiation therapy.

## Discussion

Prostate cancer is the second leading cause of death due to malignant disease in U.S. [5]. The most common sites of prostate cancer metastasis include the bone, lung and liver [6]. Brain metastasis is very rare. The reported incidence at autopsy vary from 0.6% to 4.4% and mostly involved structure are the leptomeninges (67%), followed by the cerebrum (25%) and cerebellum (8%) [6, 7]. On the other hand, the diagnosis of metastatic prostate carcinoma to the brain has rarely been made in living patients. However, with the increasing incidence of this type of cancer and with improved patient survival from better therapeutic regimens, the incidence of symptomatic brain metastasis from prostate carcinoma is likely to increase [8].

Despite prostatic neoplastic cells seem to have a low affinity for cerebral tissue [9], Sutton et al. suggest that brain metastasis appear when a severe impairment of immune system or a destruction of the blood brain barrier occur [6]. This hypothesis is confirmed by the fact that brain prostate metastasis represent often a late event when the disease has already metastasizes widespread to other organs. Routes of dissemination of advanced stage prostate cancer to the CNS include direct extension from a skull lesion, lymphatic spread and vascular embolization [10].

In all published series reviewed (Table 1), adenocarcinoma was the most common histologic type in brain metastasis from prostate carcinoma, as expected. However, other histologic types far less common at the primary site had a greater likelihood of brain metastasis than could be expected from their incidence. In particular, Salvati et al. in their series found that in patients with small cells carcinoma (SCC) the interval between diagnosis of primitive and clinical onset of brain metastasis was shorter than in the cases of adenocarcinoma [5]. Serum levels of PSA are not correlated with the development of brain metastasis, as demonstrated by literature [7, 11].

**Table 1:** Largest Series Reported in Literature and Reviewed

Authors and year of publication	Number of cases analyzed
McCutcheon et al. 1999	38
Tremont-Lukats et al. 2003	103
Po-Hui Chiang et al. 2004	13
Salvati et al. 2005	13

In our review, the most common neurological manifestations were nonfocal and included cognitive changes and headache, suggesting that the final pathogenic mechanisms resulting in clinical symptoms frequently are increased intracranial pressure and a frontal lobe syndrome. However, multifocal symptoms may be expected when the brain lesions are multiple as in the case we reported. Seizures are also pretty common and should be considered a clear sign of brain involvement.

Treatments available for intracranial metastasis include neurosurgery, external beam radiation and hormonal manipulation [6, 10]. Tremont-Lukats et al. reported that these patients, if left untreated, present a very poor survival (about 1 - 2 months). Surgery associated with whole brain radiotherapy (WBRT) seems to be effective in the control of brain lesions both relieving neurological symptoms and prolonging survival, even if prognosis remains dismal. At the light of the analyzed literature, we suggest surgery of the metastatic lesion or of the most symptomatic one in case of multiple lesions followed by WBRT. Differently, when lesions are deep or located in eloquent areas, because of the high surgical risks, a biopsy and radiotherapy should be performed.

### Conclusions

Brain metastasis from prostate carcinoma is a rare, terminal event with death in one year frequently due to the advanced systemic disease. The possibility of intraparenchymal metastasis always should be considered in a patient with prostate carcinoma who develops neurologic symptoms. On the contrary, the prostate should not be neglected as a possible source of the metastasis in male patients presenting with brain metastasis without any known site of primary tumor. Finally, a better understanding of the biology of prostate carcinoma will help clarify the basis for its metastasis to the brain.

## References

[1] Tremont-Lukats IW, Bobustuc G, Lagos GK, Lolas K, Kyritsis AP, Puduvalli VK (2003). Brain metastasis from prostate carcinoma: The M. D. Anderson Cancer Center experience. Cancer.

[2] Chiang PH, Lee TC, Huang CC (2004). Intracranial metastasis of prostate cancer: report of two cases. Chang Gung Med J.

[3] Gilles FH, Sobel EL, Tavare CJ, Leviton A, Hedley-Whyte ET (1995). Age-related changes in diagnoses, histological features, and survival in children with brain tumors: 1930-1979. The Childhood Brain Tumor Consortium. Neurosurgery.

[4] Zhang X, Tsukuda F, Yamamoto N, Takenaka I (1997). Brain metastasis from prostate cancer: a case report. Int J Urol.

[5] Salvati M, Frati A, Russo N, Brogna C, Piccirilli M, D'Andrea G, Occhiogrosso G (2005). Brain metastasis from prostate cancer. Report of 13 cases and critical analysis of the literature. J Exp Clin Cancer Res.

[6] Sutton MA, Watkins HL, Green LK, Kadmon D (1996). Intracranial metastases as the first manifestation of prostate cancer. Urology.

[7] Fervenza FC, Wolanskyj AP, Eklund HE, Richardson RL (2000). Brain metastasis: an unusual complication from prostatic adenocarcinoma. Mayo Clin Proc.

[8] McCutcheon IE, Eng DY, Logothetis CJ (1999). Brain metastasis from prostate carcinoma: antemortem recognition and outcome after treatment. Cancer.

[9] Delattre JY, Krol G, Thaler HT, Posner JB (1988). Distribution of brain metastases. Arch Neurol.

[10] Kunkler RB, Cooksey G, Millac P (1993). Carcinoma of the prostate presenting with a cerebral metastasis. Br J Urol.

[11] Erasmus CE, Verhagen WI, Wauters CA, van Lindert EJ (2002). Brain metastasis from prostate small cell carcinoma: not to be neglected. Can J Neurol Sci.

